# Influenza A virus circumvents the innate immune response through the sequestration of double-stranded RNA

**DOI:** 10.1128/jvi.00737-25

**Published:** 2025-09-08

**Authors:** Masahiro Nakano, Sho Miyamoto, Chiho Ohnishi, Chiharu Nogami, Nanami Hirose, Yoko Fujita-Fujiharu, Yukiko Muramoto, Takeshi Noda

**Affiliations:** 1 Laboratory of Ultrastructural Virology, Institute for Life and Medical Sciences, Kyoto University 12918, Kyoto, Japan; 2 Graduate School of Biostudies, Kyoto University 98344, Kyoto, Japan; 3 CREST, Japan Science and Technology Agency 12918, Saitama, Japan; 4 Department of Pathology, National Institute of Infectious Diseases 12918, Tokyo, Japan; 5 Department of Cell and Virus Structure, Max Planck Institute of Biochemistry, Martinsried, Germany; University Medical Center Freiburg, Freiburg, Germany

**Keywords:** influenza virus, innate immune evasion, double-stranded RNA, immunofluorescence assay, atomic force microscopy

## Abstract

Double-stranded RNA (dsRNA), which induces an innate immune response against viral infections, is rarely detected in influenza A virus (IAV)-infected cells. Nevertheless, we previously reported that the influenza A viral ribonucleoprotein (vRNP) complex generates looped dsRNAs during RNA synthesis *in vitro*. This finding suggests that IAV possesses a specific mechanism for sequestering dsRNA within infected cells, thereby enabling viral evasion of the innate immune response. Here, we found that dsRNAs were detected in infected cells lacking the expression of viral non-structural protein 1 (NS1) and nuclear export protein (NEP), both encoded by the same RNA segment. Indeed, the looped dsRNA-vRNP complexes were isolated from IAV-infected cells. Interestingly, NS1 molecules masked the entire looped dsRNA generated by vRNP *in vitro*, implying a potential role for NS1 in segregating viral dsRNA from cytoplasmic dsRNA sensors. Furthermore, dsRNAs were sequestered within the nucleus of wild-type IAV-infected cells, whereas their translocation to the cytoplasm was observed in NS1-deleted mutant virus-infected cells expressing M1 and NEP. This result indicates the possibility that dsRNA is transported to the cytoplasm in association with vRNP. Notably, the cytoplasmic translocation of dsRNA triggered the nuclear translocation of interferon regulatory factor 3, suggesting the capability of dsRNA in inducing the innate immune response. These findings highlight IAV’s distinctive strategy for circumventing innate immunity by sequestration of dsRNAs.

**IMPORTANCE:**

It is widely recognized that double-stranded RNA (dsRNA) produced during viral infection triggers an innate immune response. However, the influenza A virus (IAV) has been thought to rarely produce dsRNA within infected cells. Here, we detected dsRNA in the nucleus of IAV-infected cells which lacked the expression of viral non-structural protein 1 (NS1) and nuclear export protein (NEP), both encoded by a single RNA segment. High-speed atomic force microscopy demonstrated that NS1 entirely concealed dsRNA produced by the viral ribonucleoprotein complexes, thereby segregating it from cytoplasmic dsRNA sensors that trigger the innate immune response. Interestingly, cytoplasmic translocation of dsRNA was observed in cells infected with an NS1-deleted mutant virus, where M1 and NEP were expressed, resulting in the nuclear translocation of interferon regulatory factor 3. Collectively, our findings suggest that IAV adeptly sequesters dsRNA to evade the innate immune system.

## INTRODUCTION

It has long been acknowledged that double-stranded RNA (dsRNA) generated during viral infection induces an innate immune response (1–3). In non-plasmacytoid dendritic cells, viral dsRNAs are recognized by cytoplasmic dsRNA sensors, such as retinoic acid-inducible gene-I (RIG-I) and melanoma differentiation-associated gene 5 (MDA5) (4, 5). These sensors have been reported to recognize distinct cytoplasmic dsRNA species; RIG-I detects 5´-triphosphate (5´-ppp)-containing panhandle dsRNA, while MDA5 recognizes relatively long dsRNAs (6–11). Upon recognizing viral RNAs, RIG-I and MDA5 change their conformation to form filamentous oligomers on substrate RNAs and associate with mitochondrial antiviral signaling protein (MAVS) (12–14). The interaction between RIG-I/MDA5 and MAVS triggers downstream signaling pathways, ultimately resulting in the phosphorylation-driven nuclear translocation of interferon (IFN) regulatory factor (IRF) 3/7 and nuclear factor (NF)-κB. These activated factors orchestrate the transcriptional induction of type I and type III IFNs as well as proinflammatory cytokine genes (15–17).

While the majority of viruses generate dsRNA during their replication processes, it has been reported that certain negative-stranded RNA viruses, including influenza A virus (IAV), do not accumulate significant levels of dsRNA within virus-infected cells (18, 19). IAV has eight single-stranded viral genomic RNA segments (vRNAs), each of which is associated with multiple copies of a nucleoprotein (NP) and a heterotrimeric RNA-dependent RNA polymerase complex (PB2, PB1, and PA) to form a helical ribonucleoprotein complex known as the vRNP (viral ribonucleoprotein) (20–22). The transcription and replication of vRNA are conducted by the RNA polymerase complex in the context of vRNP in the nucleus of infected cells. Because nascent viral RNA is promptly separated from template vRNA following its synthesis (23), it is postulated that dsRNA intermediates are not produced during the replication process of IAV. Consequently, in IAV-infected cells, the induction of the innate immune response is thought to be triggered by the 5′-ppp panhandle structure of vRNA (11, 24–26), defective viral genomes (27), or short aberrant vRNAs (28).

In contrast, we recently found that vRNP isolated from IAV virions frequently generates looped dsRNAs *in vitro* (29). Notably, the vRNP associated with these looped dsRNAs underwent substantial deformation, adopting a nonhelical configuration. This phenomenon led us to speculate that dsRNA formation represents an aberration in RNA synthesis, in which nascent viral RNA fails to separate from the template vRNA. Additionally, we detected dsRNA in IAV-infected cells; however, the proportion of cells producing dsRNA was minimal (0.16% of infected cells) (29). This finding suggests that IAV employs a specialized mechanism to sequester dsRNA within infected cells, thereby circumventing the innate immune response. In the present study, we aimed to determine how IAV conceals dsRNA in infected cells, shedding light on the strategies employed to evade immune responses.

## RESULTS

### dsRNA is detected in a limited number of IAV-infected cells

**Fig 1:**
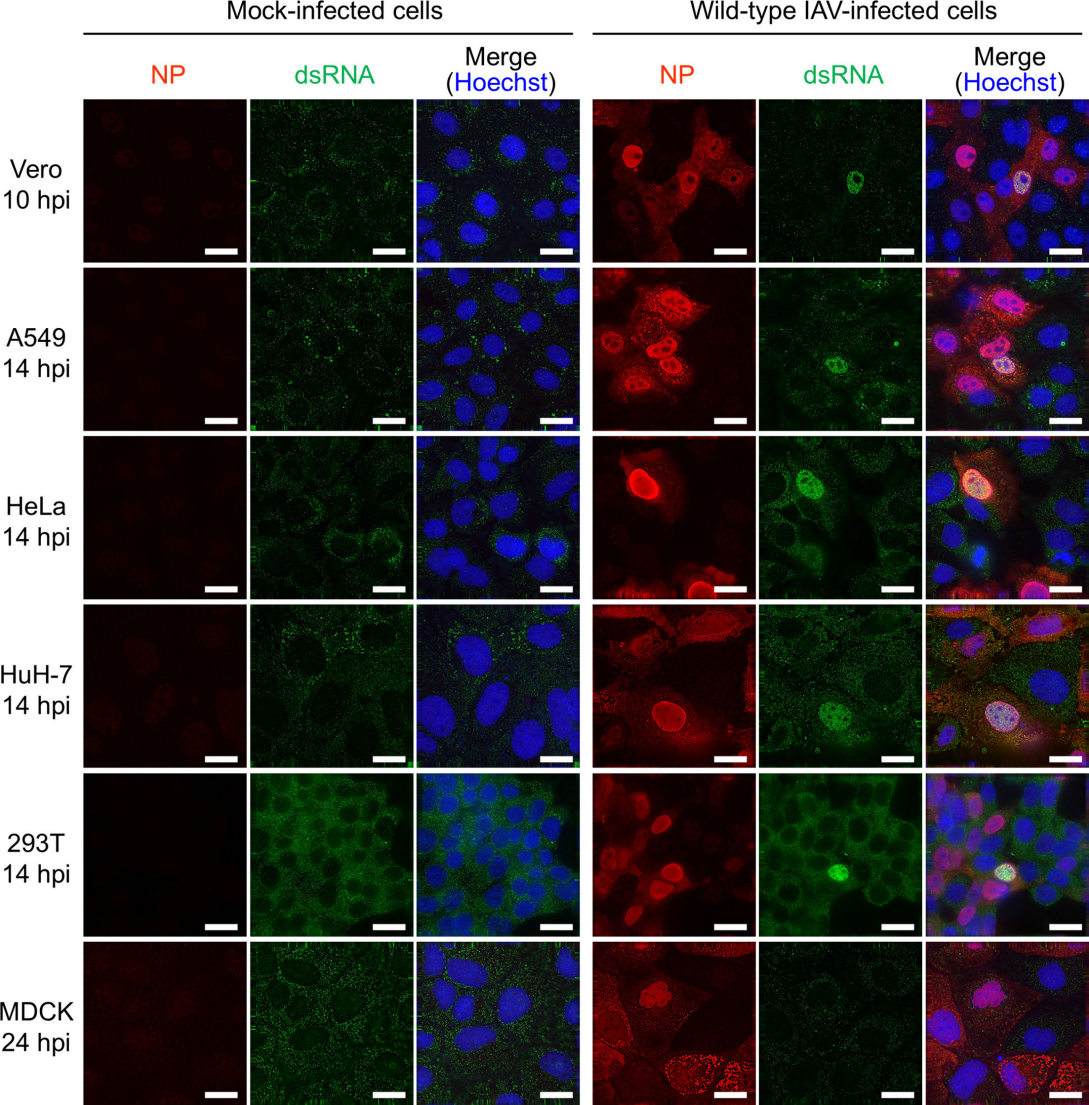
dsRNA detection in IAV-infected cells. Cells subjected to mock infection (left panels) or infected with the IAV WSN strain at an MOI of 0.1 (right panels) were fixed at 10 hpi for Vero cells; 14 hpi for A549, HeLa, HuH-7, and 293T cells; or 24 hpi for MDCK cells. The presence of NP and dsRNA in infected cells was determined by IFA using anti-NP and anti-dsRNA antibodies, respectively. Cell nuclei were stained with Hoechst. The scale bars represent 20 µm.

Previous studies indicated that IAV does not produce detectable amounts of dsRNA in infected cells (18, 19). In contrast, we previously found that dsRNA was detected within the nuclei of Vero cells infected with the influenza A/Puerto Rico/8/34 (H1N1) (PR8) virus (29). To assess dsRNA detection across diverse cell lines, we infected various cell types with influenza A/WSN/33 (H1N1) virus at a multiplicity of infection (MOI) of 0.1. Immunofluorescence assays (IFAs) using an anti-dsRNA antibody showed the absence of dsRNA in mock-infected cell lines (Fig. 1, left panels). In IAV-infected Vero cells, dsRNA was detected in the nucleus at 10 h post-infection (hpi) (Fig. 1, right panels), in line with our previous study employing the PR8 virus (29). Beyond 14 hpi, dsRNA was detected in the IAV-infected A549, HeLa, HuH-7, and 293T cells (Fig. 1, right panels). It should be noted that dsRNA was exclusively detected in the nucleus, with no detectable presence in the cytoplasm of these cell lines. Importantly, dsRNA detection was limited to a small number of infected cells (approximately 0.1% of infected cells), as previously documented in our study using Vero cells (29). Contrary to our expectations, dsRNA was not detected in IAV-infected Madin-Darby canine kidney (MDCK) cells even at 24 hpi (Fig. 1, right panels), although the underlying reason remains unknown. These findings indicate that, while dsRNAs can be detected in various cell types upon IAV infection, their detection occurs under specific conditions.

### dsRNA is detected in IAV-infected cells lacking non-structural protein 1 (NS1) and nuclear export protein (NEP) expression

**Fig 2:**
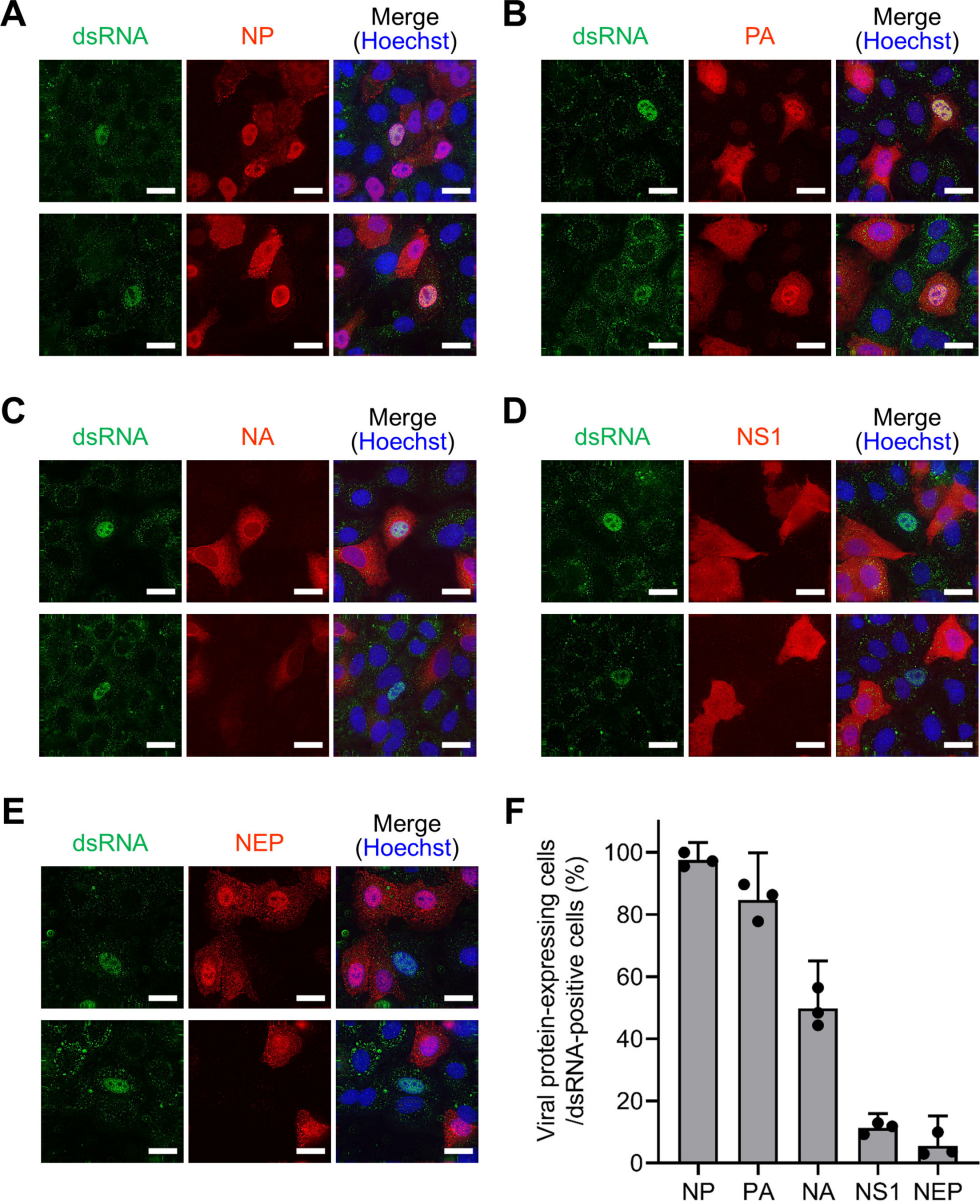
Viral protein expression in dsRNA-positive cells. A549 cells were infected with the IAV WSN strain at an MOI of 0.1 and subsequently fixed at 14 hpi. The presence of viral proteins in infected cells was detected by IFA using antibodies against NP (**A**), PA (**B**), NA (**C**), NS1 (**D**), and NEP (**E**). Additionally, an anti-dsRNA antibody was used to detect dsRNA, while Hoechst was used to stain the cell nuclei. For each panel (**A–E**), two representative image sets (top and bottom) from a single experiment are shown. The experiment was independently repeated at least three times with similar results. The scale bars denote 20 µm. (**F**) The percentage of dsRNA-positive cells expressing each viral protein was calculated from the IFA results. The presented data represent the mean with 95% confidence intervals derived from three biologically independent experiments. Detailed counts of viral protein-expressing and dsRNA-positive cells are presented in Table S2.

To understand the restrictive nature of dsRNA detection within IAV-infected cells, the conditions for dsRNA detection were explored. Vero and A549 cells were infected with IAV at different MOI, and dsRNAs were detected by IFA. Intriguingly, dsRNAs were barely observable at a high MOI (MOI = 5), whereas they were detected in infected cells at a low MOI (MOI = 0.1) (Fig. S1 and Table S1). Considering the established knowledge that IAV virions fail to express one or more viral proteins under low MOI conditions (30, 31), we speculated that the expression of the viral protein responsible for preventing dsRNA detection was lacking in dsRNA-positive infected cells at a low MOI. Therefore, we investigated the relationship between dsRNA detection and viral protein expression at a low MOI. Upon infecting A549 cells with IAV at an MOI of 0.1, NP and PA, which are integral components of vRNA replication, were ubiquitously expressed within the dsRNA-positive cells (Fig. 2A, B, and F and Table S2). Neuraminidase (NA), a spike protein present in the viral envelope, was detected in 50% of dsRNA-positive cells (Fig. 2C and F and Table S2). In contrast, two viral proteins originating from RNA segment 8, NS1 and NEP, exhibited limited expression in dsRNA-positive cells (Fig. 2D through F and Table S2). Simultaneous detection revealed the absence of both NS1 and NEP in these cells (Fig. S2). These findings suggest that IAV generates dsRNA through vRNP during replication while concurrently concealing dsRNA via NS1 and/or NEP.

### IAV conceals looped dsRNA through the utilization of NS1

**Fig 3:**
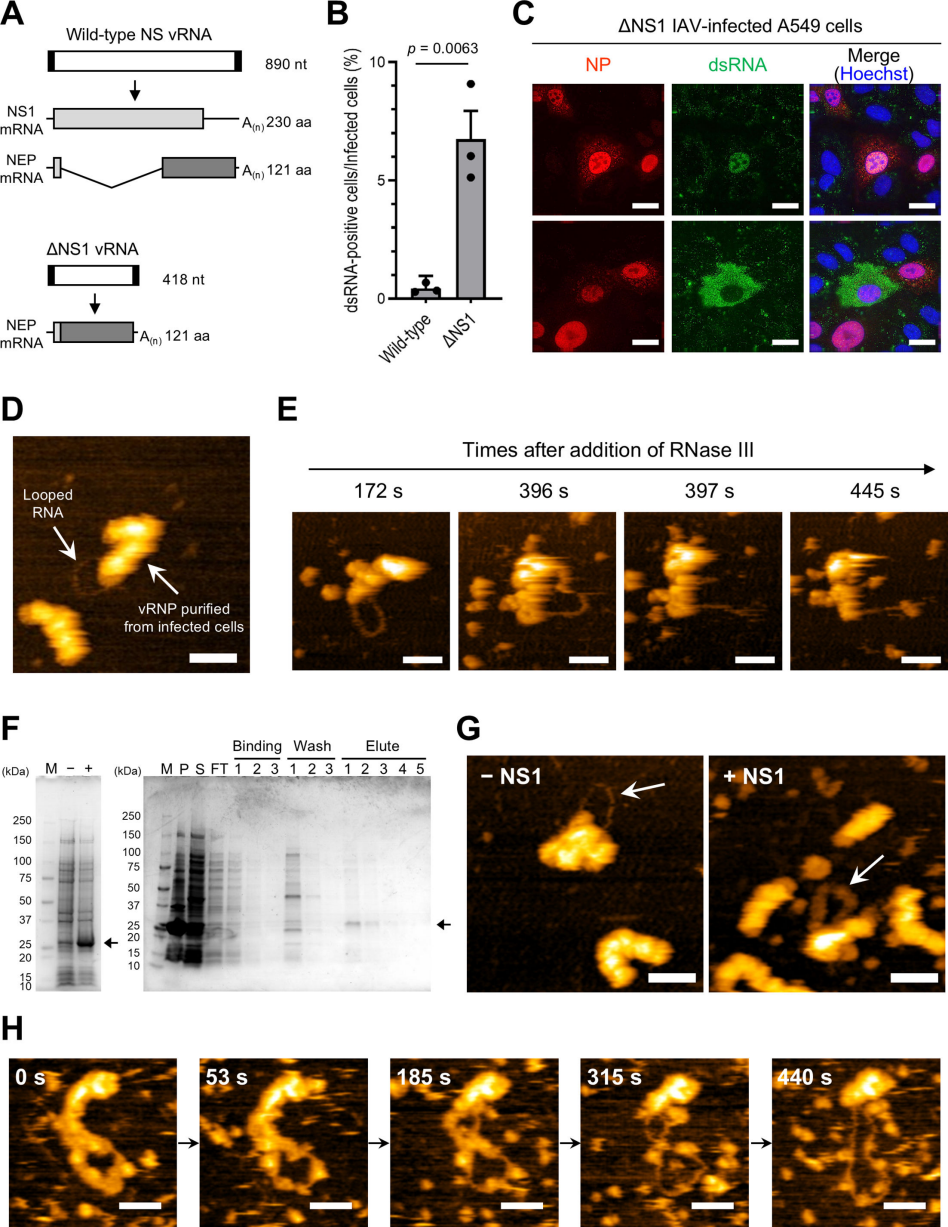
Concealment of dsRNA by NS1. (**A**) Schematic of NS genes and NS-specific mRNAs. A mutant NS gene which lacks NS1 open reading frame was designed, as described by Garcia-Sastre et al. (35). Both NS1 and NEP mRNA are synthesized from wild-type NS vRNA (upper panel), whereas only NEP mRNA is transcribed from ΔNS1 vRNA (lower panel). The vRNA segments are depicted as white boxes flanked by black boxes, which denote the non-coding regions of the gene. The coding sequence of the NS1 protein is shown as a light gray box, whereas that of NEP is shown as a dark gray box. NEP mRNA originating from the wild-type NS vRNA is a spliced product of the NS1 mRNA, as shown by the V-shaped line. (**B**) Comparison of dsRNA production in wild-type IAV and ΔNS1 virus infections. A549 cells were infected with wild-type IAV or ΔNS1 virus at an MOI of 0.1 and were fixed at 24 hpi. By IFA, the percentage of dsRNA-positive cells within infected cells was calculated. The presented data reflect the average ± 95% confidence intervals from three biologically independent experiments. Significance was determined using an unpaired *t*-test. (**C**) dsRNA production in cells infected with the ΔNS1 virus. A549 cells were infected with the ΔNS1 virus at an MOI of 0.1 and were fixed at 14 hpi. The presence of NP and dsRNAs was detected via IFA using anti-NP and anti-dsRNA antibodies, respectively. Cell nuclei were stained with Hoechst. Representative images of cells producing dsRNAs within the nucleus (upper panels) and cytoplasm (lower panels), from different fields of view in a single experiment, are shown. The experiment was independently repeated at least three times with comparable results. Scale bars: 20 µm. (**D**) HS-AFM observation of vRNPs purified from FLAG–PB2 virus-infected cells. The looped RNA and its associated vRNP are indicated by arrows. (**E**) Digestion of looped RNA by RNase III. During the HS-AFM observation of vRNPs purified from FLAG–PB2 virus-infected cells, RNase III was introduced, and digestion of the looped RNA was monitored. Four representative images captured at designated intervals are presented, displaying digestion occurring between 396 and 397 s. (**F**) Generation of recombinant NS1 protein. (Left panel) Expression of recombinant NS1 protein. *Escherichia coli* cellular precipitates, both pre-induction (−) and post-induction (+) with isopropylthio-β-galactoside, underwent SDS-PAGE followed by Coomassie brilliant blue (CBB) staining. The band corresponding to expressed NS1 is denoted by an arrow. (Right panel) Purification of recombinant NS1 protein. *E. coli* expressing NS1 was subjected to sonication and centrifugation. The resulting precipitate (P) and supernatant (S) were analyzed via SDS-PAGE followed by CBB staining to ascertain the acquisition of recombinant NS1 as a soluble protein. Subsequently, the supernatant was subjected to purification using a Ni^2+^-immobilized column. After washing the column with binding and wash buffer, the recombinant NS1 was eluted using elute buffer, as indicated by the arrow. Molecular weight marker proteins (M) were utilized for reference. The flow-through of the affinity column is labeled as FT. Uncropped gel images are shown in Fig. S8. (**G**) Masking looped RNA via NS1. Following *in vitro* RNA synthesis using vRNPs purified from IAV virions, the recombinant NS1 protein was introduced. The mixture without (left panel) and with NS1 (right panel) was visualized using HS-AFM, with the looped structure in each image indicated by an arrow. (**H**) Detachment of NS1 from the looped RNA. The thick-looped structure observed after NS1 addition was monitored over an extended duration using HS-AFM, applying an augmented force. Five representative images captured at specific intervals are presented. All scale bars in the HS-AFM images are 50 nm.

Because NS1 and NEP are absent in dsRNA-positive cells, it was postulated that IAV conceals dsRNA through NS1 and/or NEP. NS1 acts as an antagonist of type I IFN and exhibits dsRNA-binding capabilities (32–34). Accordingly, we focused on NS1 and prepared a mutant IAV lacking the NS1 gene (ΔNS1 virus) (35) to examine the role of NS1 in concealing dsRNA. As illustrated in Fig. 3A, the ΔNS1 virus exclusively produces NEP from the truncated vRNA, while the wild-type IAV expresses both NS1 and NEP from the NS vRNA segment. To compare dsRNA detection between ΔNS1- and wild-type virus-infected cells, A549 cells were infected with both viruses at the same MOI (MOI = 0.1), followed by IFA. The proportion of dsRNA-positive cells among infected cells was 15 times higher in ΔNS1 virus-infected cells than in wild-type virus-infected cells (Fig. 3B), implying that NS1 bound to dsRNA and prevented its detection in wild-type virus-infected cells. Interestingly, the ΔNS1 virus-infected cells exhibited a distinctive pattern; dsRNAs were detected not only in the nucleus but also in the cytoplasm of the infected cells (Fig. 3C), which is different from the observation in wild-type virus-infected cells where dsRNA was detected only in the nucleus (Fig. 1 and 2).

Given that the vRNP purified from the IAV virion generated a looped dsRNA *in vitro* (29), we hypothesized that looped dsRNA is also generated in infected cells but is masked by NS1. To address this, we examined whether the vRNP associated with looped dsRNA was isolated from infected cells using the FLAG–PB2 WSN virus, which expresses the FLAG-tagged PB2 protein at its N-terminus (36). A549 cells were infected with this recombinant virus, and FLAG-tagged vRNPs were obtained from the infected cells at 24 hpi by immunoprecipitation and density gradient ultracentrifugation (Fig. S3). High-speed atomic force microscopy (HS-AFM) analysis revealed the presence of vRNP associated with looped RNA structures (Fig. 3D), similar to those produced through *in vitro* RNA synthesis (29). Furthermore, the looped RNA was digested by RNase III (Fig. 3E and Movie S1), indicating that the looped RNA isolated from the infected cells was indeed dsRNA produced by the vRNP. Although the number is small, the looped dsRNA-vRNP complex was consistently isolated from IAV-infected cells, providing strong evidence that IAV generates dsRNA within infected cells. However, contrary to our expectations, only naked-looped dsRNA was isolated, and dsRNA masked by NS1 was not observed in the present study.

Subsequently, we investigated whether the recombinant NS1 protein could mask the dsRNA generated by the vRNP *in vitro*. Following *in vitro* RNA synthesis using purified vRNP, the purified recombinant NS1 protein (Fig. 3F) was introduced, and the mixture was analyzed by HS-AFM. Remarkably, upon NS1 addition, thick-looped structures were observed (Fig. 3G and Fig. S4). The looped structures had lower heights (~3.5 nm) than the vRNP and displayed uniform widths (~25 nm) (Fig. S4). Prolonged HS-AFM observations (~5 min) with the application of increased force revealed that individual particles sequentially detached from the looped structure, exposing the naked RNA (Fig. 3H and Movie S2). These findings strongly indicate that NS1 masks looped dsRNA generated by vRNP, a phenomenon that likely occurs within infected cells.

### dsRNA undergoes translocation to the cytoplasm in association with VRNP

**Fig 4:**
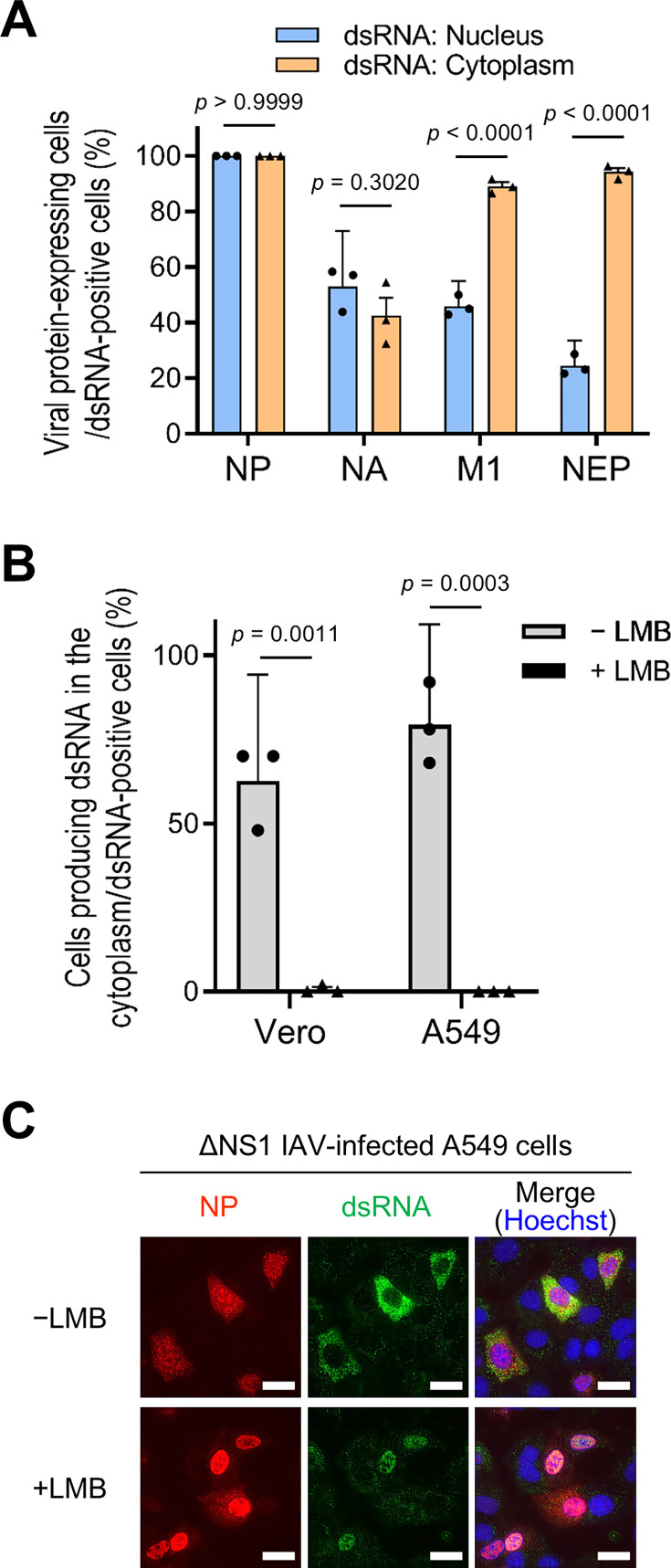
Cytoplasmic translocation of dsRNA in association with vRNP. (**A**) Relationship between dsRNA localization and viral protein expression in ΔNS1 virus-infected cells. A549 cells were infected with ΔNS1 virus at an MOI of 0.1 and fixed at 14 hpi. The production of dsRNA and expression of each viral protein were detected by IFA, as illustrated in Fig. S5. Percentages of cells expressing each viral protein in cells producing dsRNA within the nucleus (blue) or in the cytoplasm (orange) were calculated from the IFA results. Approximately 50 dsRNA-positive cells were examined in each experiment, and the data represent the average ± 95% confidence intervals of three biologically independent experiments. Significance was determined using two-way analysis of variance with Tukey’s test. (**B and C**) Inhibition of dsRNA nuclear export by leptomycin B (LMB). Vero or A549 cells were infected with ΔNS1 virus at an MOI of 0.1 and subsequently subjected to incubation with or without LMB. The cells were fixed at 24 hpi, and dsRNA was detected by IFA. The subcellular localization of dsRNA was examined both in the absence (gray) and presence (black) of LMB. The computation of the proportions of cells producing dsRNA in the cytoplasm relative to the entirety of dsRNA-positive cells is delineated in B. In each experiment, approximately 50 cells generating dsRNA were examined, and the presented data delineate the mean ± 95% confidence intervals acquired from three biologically independent experiments. Significance was determined by multiple unpaired *t*-test. Representative A549 cell images obtained by IFA employing anti-NP and anti-dsRNA antibodies are displayed in C. Scale bars: 20 µm.

As shown in Fig. 3C, within the ΔNS1 virus-infected cell, dsRNA was detected not only in the nucleus but also in the cytoplasm. Considering that the looped dsRNA associated with vRNP was isolated from infected cells (Fig. 3D), we hypothesized that the cytoplasmic detection of dsRNA originated from the initial generation of dsRNA within the nucleus, followed by subsequent translocation to the cytoplasm. To investigate the potential nuclear export of dsRNA, we sought to distinguish between cells in which dsRNA is detected in the nucleus and those in which dsRNA is detected in the cytoplasm. Therefore, the expression of viral proteins in ΔNS1 virus-infected dsRNA-positive A549 cells was examined. When dsRNA was detected in the nucleus, the expression of viral proteins, excluding NP, was verified in 25%–53% of dsRNA-positive cells, indicating that these viral proteins—NA, M1, and NEP—were dispensable for dsRNA detection (Fig. 4A and Fig. S5). Conversely, when dsRNA was identified in the cytoplasm, the expression of the two viral proteins underwent substantial alterations; M1 and NEP were expressed in nearly all dsRNA-positive cells, whereas the expression of NA did not change significantly (Fig. 4A and Fig. S5). Intriguingly, both M1 and NEP are known for their pivotal roles in the nuclear export of vRNP (37–39), suggesting the possibility that dsRNAs are transported from the nucleus to the cytoplasm along with vRNP, mediated by these viral proteins.

Considering that dsRNA appears to be transported to the cytoplasm through its association with vRNP, we investigated whether inhibiting vRNP nuclear export would diminish the cytoplasmic localization of dsRNA. Vero and A549 cells were infected with the ΔNS1 virus in the presence or absence of leptomycin B (LMB), a known inhibitor of the nuclear export of vRNP via CRM1 (40), and the localization of dsRNA was assessed using IFA. In the absence of LMB, approximately 60% and 80% of dsRNA-positive Vero and A549 cells, respectively, exhibited cytoplasmic localization of dsRNA (Fig. 4B and C). In contrast, in the presence of LMB, cytoplasmic localization was significantly reduced in both Vero and A549 cells, with only approximately 1% of dsRNA-positive cells displaying cytoplasmic localization of dsRNA. While LMB inhibits the nuclear export of newly synthesized vRNPs and can reduce subsequent rounds of infection, it had no significant impact on the percentage of infected or dsRNA-positive cells (Fig. S6), suggesting that the majority of infected cells observed in our experiments likely originated from the input inoculum rather than from subsequent rounds of viral replication. These results strongly indicate that the dsRNA detected in IAV-infected cells is produced by vRNP in the nucleus and is exported to the cytoplasm in association with the vRNP.

### IAV induces innate immune response upon the export of dsRNA to the cytoplasm

**Fig 5:**
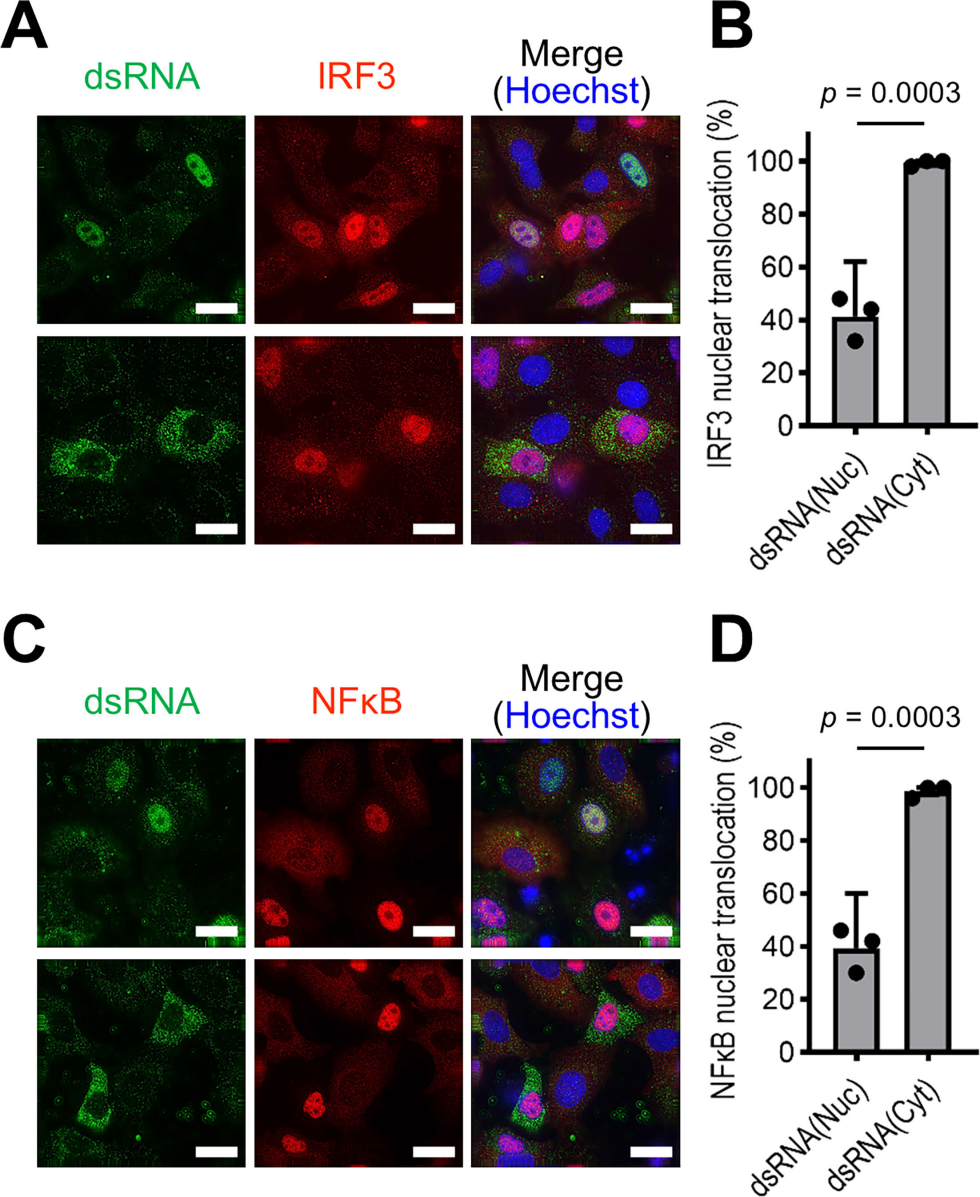
Induction of the innate immune response by dsRNA. A549 cells were infected with ΔNS1 virus at an MOI of 0.2 and were fixed at 24 hpi. Antibodies against IRF3 (**A and B**) and NFκB (**C and D**) were employed in IFA for the identification of nuclear translocation of IRF3 and NFκB, respectively. The cells were fixed at 24 hpi and stained with these antibodies. Representative image sets of cells producing dsRNAs within the nucleus (upper panels) and cytoplasm (lower panels), from different fields of view in a single experiment, are shown in A and C. All scale bars denote 20 µm. The percentage of cells exhibiting nuclear translocation of each transcription factor within cells producing dsRNA in the nucleus (Nuc) or cytoplasm (Cyt) is shown in **B** and **D**. For each enumeration, 50 dsRNA-positive cells were examined, and the data represent the mean ± 95% confidence intervals acquired from three biologically independent experiments. Statistical significance was determined by unpaired *t*-test.

dsRNAs induce innate immune responses in virus-infected cells (1–3). Our findings suggest that dsRNA detected in IAV-infected cells, upon translocation to the cytoplasm, is recognized by cytoplasmic dsRNA sensors such as RIG-I and MDA5. Therefore, to ascertain whether the dsRNA was indeed responsible for eliciting the innate immune response, dsRNA detection and IRF3 nuclear translocation in each cell line were analyzed using IFA. Upon infecting A549 cells with wild-type IAV, dsRNAs were exclusively detected within the nucleus, and nuclear translocation of IRF3 was not associated with dsRNA detection (Fig. S7A). In ΔNS1 virus-infected cells, the nuclear translocation of IRF3 remained unaffected by dsRNA detection when dsRNA was localized in the nucleus; the nuclear translocation of IRF3 was observed in approximately 40% of the cells in which dsRNA was detected in the nucleus (Fig. 5A, upper and B). Conversely, when dsRNA was detected in the cytoplasm, nearly all dsRNA-positive cells exhibited nuclear translocation of IRF3 (Fig. 5A, lower and B). Furthermore, quadruple staining for dsRNA, viral NP, IRF3, and the nucleus revealed that approximately 98% of cells exhibiting IRF3 nuclear translocation were NP-positive, indicating that this translocation occurs exclusively in infected cells (Fig. S7B). Similar results were observed for the nuclear translocation of NF-κB (Fig. 5C and D). It should be noted that a substantial number of dsRNA-negative cells exhibited IRF3/NF-κB nuclear translocation, suggesting that the nuclear translocation was induced not only by cytoplasmic dsRNA but also by undetectable RNA species, such as the 5′-ppp panhandle structure of vRNA (11, 24–26), defective viral genomes (27), and short aberrant vRNAs (28), which are previously reported to induce the innate immunity. Nevertheless, our results suggest that the cytoplasmic localization of dsRNA induces the innate immune response, whereas nuclear localization of dsRNA does not necessarily elicit such a response.

## DISCUSSION

In the present study, we demonstrated that dsRNA is detected within IAV-infected cells, wherein the expression of NS1 is notably absent. IAV appears to strategically utilize NS1 to conceal dsRNA, thereby segregating it from cytoplasmic dsRNA sensors such as RIG-I and MDA5. Indeed, upon translocation into the cytoplasm, the dsRNA induces the nuclear translocation of IRF3. These findings imply that IAV employs a distinctive strategy to circumvent innate immune responses.

In IAV-infected cells, production of dsRNA is usually suppressed by RNA polymerase and host factors such as UAP56, thus nascent viral RNA is promptly separated from template vRNA during replication (23, 41). However, here, we suggest that IAVs aberrantly generate dsRNAs in diverse cell types, although the occurrence of dsRNA-positive cells is limited (Fig. 1). Previous studies have shown that dsRNAs are rarely detected in IAV-infected cells (18, 19). In these investigations, cells were infected with a relatively high MOI of IAV (MOI = 3–5), whereas we revealed the significance of a low MOI in facilitating dsRNA generation (Fig. S1 and Table S1). IAV infection at a low MOI yields infected cells that lack one or more viral proteins (30, 31). Although the mechanism governing the generation of virions that express an incomplete set of viral proteins remains unclear, it appears to occur when virions fail to package one or more vRNA segments or when incoming vRNPs undergo unsuccessful nuclear import, transcription, and/or replication. It was suggested that IAV-infected cells expressing NP and RNA polymerases but lacking NS1 are unable to mask the dsRNA generated by vRNP, leading to the detection of dsRNA within the infected cells. Conversely, in cells expressing the complete repertoire of viral proteins, if dsRNA is aberrantly generated during RNA synthesis, IAV concurrently conceals it with NS1, thereby precluding the detection of dsRNA.

The absence of NS1 in dsRNA-positive cells led us to hypothesize that NS1 masks dsRNA expression in IAV-infected cells. It has been previously reported that NS1 forms a tubular structure in the presence of dsRNA which comprises complementary strands with GA and CU repetitions (34); however, the capability of NS1 to mask dsRNA generated by IAV remains unrevealed. We previously found that vRNP purified from the IAV virion produced a looped dsRNA *in vitro* (29). Therefore, we aimed to purify the looped dsRNA-vRNP complex from IAV-infected cells, wherein dsRNA was expected to be masked by NS1. Consistent with our expectations, the looped dsRNA-vRNP complex was isolated from IAV-infected cells, providing strong evidence that IAV generates dsRNA within infected cells. However, solely the naked-looped dsRNA-vRNP complexes were purified, and looped dsRNA associated with NS1 was not observed (Fig. 3D). This might be because NS1 molecules bound to dsRNA were dissociated during the purification process using detergent. In contrast, we observed the NS1 masking of looped dsRNAs produced by *in vitro* RNA synthesis. The looped dsRNA masked by NS1 exhibited regular width and height (Fig. 3G and Fig. S4), suggesting that NS1 molecules were uniformly associated with the dsRNA. While NS1 molecules detached from the dsRNA during HS-AFM observation, they required a prolonged duration and substantial applied force for dissociation, implying that NS1 strongly masks dsRNA in infected cells. These findings suggest that IAV employs NS1 to conceal dsRNA, thereby evading the innate immune response.

**Fig 6:**
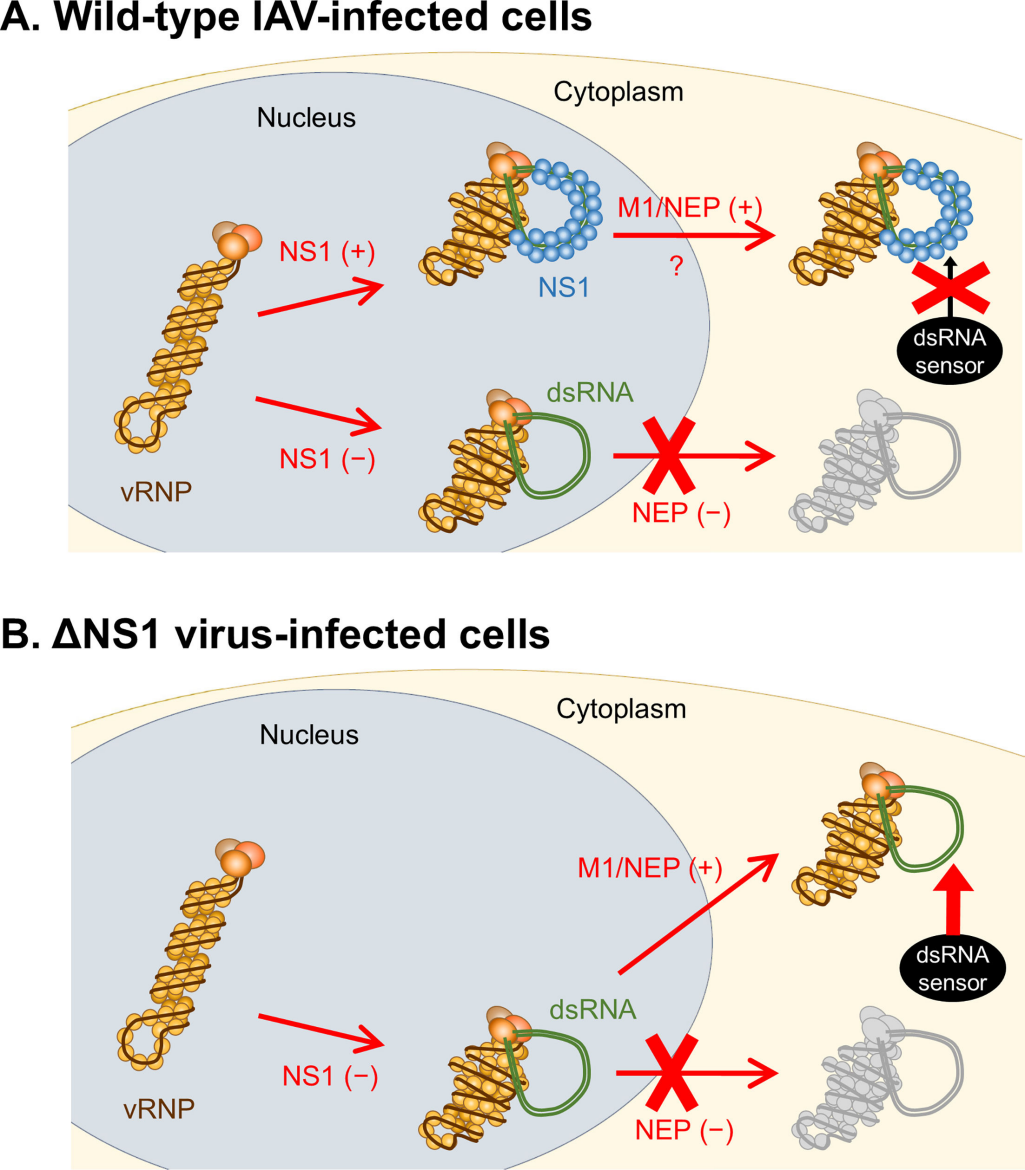
Proposed strategies for circumventing the innate immune response employed by IAV. (**A**) dsRNA production in the wild-type IAV-infected cells. When NS1 is expressed (upper), dsRNA aberrantly generated by vRNP is concurrently masked by NS1. Although the translocation of masked dsRNA to the cytoplasm remains undetermined, cytoplasmic dsRNA sensors appear to be incapable of accessing IAV dsRNA, thereby enabling IAV to circumvent the innate immune response. Conversely, in scenarios where NS1 expression is absent (lower), a detectable amount of dsRNA is generated within the nucleus. Nevertheless, in the absence of NS1 expression, NEP expression is also absent, leading to the sequestration of dsRNA within the nucleus and, consequently, the isolation of dsRNA from the cytoplasmic dsRNA sensors. (**B**) dsRNA production in the ΔNS1 virus-infected cells. When dsRNA is aberrantly produced by vRNP, it remains unmasked due to the absence of NS1. The unmasked dsRNA is transported to the cytoplasm in infected cells expressing M1 and NEP, leading to its recognition by RNA sensors and subsequent induction of the innate immune response. In these panels, only vRNPs responsible for dsRNA production are illustrated. vRNPs that produce normal viral RNAs (mRNA and vRNA) or do not engage in RNA synthesis are omitted for clarity.

The present study using the ΔNS1 virus indicates that dsRNA is transported to the cytoplasm in association with vRNP, as this translocation was observed in infected cells expressing M1 and NEP, both of which are essential for the nuclear export of vRNP (37–39). Moreover, we identified that the dsRNA triggers the nuclear translocation of IRF3/NF-κB upon its translocation to the cytoplasm (Fig. 5). Although further studies are required to ascertain whether the dsRNA is indeed recognized by cytoplasmic dsRNA sensors such as RIG-I and MDA5, these results suggest the potential of the dsRNA to induce an innate immune response. On the other hand, IAV appears to ingeniously segregate dsRNA from cytoplasmic RNA sensors by masking the entirety of dsRNA with NS1. In wild-type IAV-infected cells expressing NS1, the translocation of masked dsRNA to the cytoplasm remains uncertain; nevertheless, it will evade recognition by RNA sensors, given their incapacity to access the shielded dsRNA (Fig. 6A, upper). In contrast, in wild-type IAV-infected cells devoid of NS1 expression, detectable amounts of dsRNA are aberrantly generated. However, in such instances, NEP expression is simultaneously absent because both NS1 and NEP are encoded by the same RNA segment, with NEP expressed from the spliced NS mRNA (Fig. 3A). Accordingly, in the context of wild-type virus infection, cells that lack NS1 but produce NEP would not be observed, causing the dsRNA to remain within the nucleus (Fig. 6A, lower). Conversely, in ΔNS1 virus-infected cells, aberrantly produced dsRNA is unmasked due to the absence of NS1. The unmasked dsRNA is transported to the cytoplasm in the presence of M1 and NEP, leading to its recognition by cytoplasmic RNA sensors (Fig. 6B). A similar situation may also occur in wild-type IAV-infected cells if NS1 fails to mask dsRNA and the unmasked dsRNA is transported to the cytoplasm by M1 and NEP. It has been documented that a singular dsRNA molecule alone possesses the capability to elicit the innate immune response (42). Consequently, there is a potential scenario wherein undetectable amounts of dsRNA in the cytoplasm prompt the nuclear translocation of IRF3/NF-κB. For instance, in the current investigation, IRF3/NF-κB nuclear translocation was observed even when dsRNA was not detected in the cytoplasm (Fig. 5), suggesting the possibility that this nuclear translocation was induced by undetectable cytoplasmic dsRNA. Nevertheless, given that the proportion of IRF3 nuclear translocation when dsRNA was detected in the nucleus was approximately 40%, it appears that IAV adeptly sequesters dsRNA from cytoplasmic dsRNA sensors by retaining it in the nucleus.

Collectively, we propose a distinctive strategy employed by IAV to circumvent the innate immune responses. Although only two laboratory-adapted strains (WSN and PR8) were used in this study, the dsRNA-binding activity of NS1 is mediated by highly conserved residues within the N-terminal RNA-binding domain (e.g., Arg38, Lys41), which are well preserved across diverse IAV strains and even in influenza B viruses (33). Therefore, the mechanism of dsRNA masking by NS1 observed in this study is likely to be shared by other IAVs. Nevertheless, we acknowledge that variations in expression levels and host factor interactions among different IAV strains could influence the extent of dsRNA masking. Further investigation using contemporary and diverse strains will be necessary to fully generalize our findings. Considering that dsRNA-mediated innate immune activation is a universal feature of virus infection, our results may provide insights not only into IAV pathogenesis but also into host antiviral responses against other viruses.

## MATERIALS AND METHODS

### Cells

African green monkey kidney Vero cells (CCL-81; ATCC, Manassas, VA, USA) were grown in Eagle’s minimum essential medium (MEM; Gibco, Gaithersburg, MD, USA) supplemented with 10% fetal calf serum (FCS; Biosera, Nuaille, France). Human lung carcinoma A549 cells (CCL-185; ATCC), human cervical carcinoma HeLa cells (JCRB9004; JCRB Cell Bank, Tokyo, Japan), human hepatoma HuH-7 cells (JCRB0403; JCRB Cell Bank), and human embryonic kidney (HEK) 293T cells (CRL-3216; ATCC) were cultured in Dulbecco’s modified Eagle’s medium (DMEM; Merck, Darmstadt, Germany) with 10% FCS. MDCK cells were kindly provided by Y. Kawaoka (University of Tokyo) and grown in MEM supplemented with 5% newborn calf serum (Thermo Fisher Scientific, Waltham, MA, USA). All cell cultures were maintained at 37°C in a 5% CO_2_ atmosphere.

### Plasmid construction

The NS1 deletion plasmid, denoted as pPolI-ΔNS1, was constructed following previously outlined procedures (35). For the excision of the NS1 gene, an inverse PCR was performed, utilizing pPolI-NS1 (Provided by Prof. Kawaoka, University of Tokyo) as a template, along with a primer set: 5′-GACATACTGATGAGGATGTC-3′ (delNS1_WSN_F) and 5′- CTGAAAGCTTGACACAGTGTTTG-3′ (delNS1_WSN_R). The resulting PCR product underwent DpnI treatment, purification through the MinElute PCR purification kit (Qiagen, Hilden, Germany), and subsequent ligation using T4 polynucleotide kinase (Takara, Kusatsu, Japan) and T4 DNA ligase (Ligation mix #6023; Takara). The pPolI- FLAG–PB2 plasmid was designed to generate FLAG–PB2 RNA, wherein the sequence encoding the FLAG peptide (DYKDDDDK) was fused to the sequence encoding the N-terminus of PB2 (36). Construction of the plasmid involved the insertion of the FLAG–PB2 open reading frame with a stop codon into a truncated pPolI-PB2 plasmid with the 3′ and 5′ non-coding regions.

### Generation of recombinant viruses by reverse genetics

Reverse genetics was performed using pPolI plasmids encompassing the cDNA sequences of influenza A/WSN/1933 (WSN; H1N1) viral genes positioned between the human PolI promoter and the mouse PolI terminator, as previously outlined (43). The FLAG–PB2 virus was generated by substituting pPolI-PB2 (wild-type) with the pPolI-FLAG–PB2 plasmid. The ΔNS1 virus was generated by replacing pPolI-NS (wild-type) with the pPolI-ΔNS1 plasmid. Additionally, pPCAGGS-NS1 was co-transfected into 293T cells. The viral supernatant was inoculated into Vero cells for viral replication. The inoculated cells were cultivated in MEM/BSA at 37°C for 5 days, with the periodic addition of 1 µg/mL of TPCK trypsin at 24 h intervals (44). The resultant viral supernatant was harvested, and NS1 gene deletion was verified by reverse-transcription PCR and sequencing.

### IFA

Cells were cultured on a 35 mm glass-bottom dish (Matsunami Glass, Osaka, Japan) pre-coated with rat collagen type I (Corning, Corning, NY, USA) 1 day before infection. Subsequently, cells were infected with IAV (wild-type, ΔNS1, and FLAG–PB2 viruses) at an indicated MOI, followed by incubation for specified durations in MEM/BSA. In experiments concerning the inhibition of vRNP nuclear export, 5 ng/mL of leptomycin B (Cell Signaling Technology, Danvers, MA, USA) was introduced into the medium. After infection, the cells were fixed in 4% paraformaldehyde (Nacalai Tesque, Kyoto, Japan) for 10 min, followed by permeabilization with 0.1% Triton X-100 in phosphate-buffered saline (PBS) for 10 min. After washing with PBS, the cells were blocked with Blocking One (Nacalai Tesque) for 30 min. Following the blocking step, cells were incubated with anti-NP rabbit polyclonal (1:1,000 dilution, GTX125989; GeneTex, Irvine, CA, USA) and anti-dsRNA mouse monoclonal antibodies J2 (1:500 dilution, 10010200; Scicons; Nordic-MUbio, Susteren, The Netherlands) overnight at 4°C. Rabbit polyclonal antibodies against PA (1:1,000 dilution, GTX125932; GeneTex), NS1 (1:1,000 dilution, GTX125990; GeneTex), NEP (1:1,000 dilution, GTX125953; GeneTex), NA (1:1,000 dilution, GTX125974; GeneTex), and M1 (1:1,000 dilution, GTX125928; GeneTex) were used instead of the anti-NP antibody to detect IAV proteins. For the assessment of nuclear translocation of IRF3 and NF-κB, rabbit monoclonal antibodies against IRF3 (1:1,000 dilution, 11904S; Cell Signaling Technology) and NF-κB (1:1,000 dilution, 8242S; Cell Signaling Technology) were employed instead of the anti-NP antibody, respectively. Following PBS washes, cells were incubated with Alexa Fluor 488-conjugated anti-mouse antibody (1:1,000 dilution, A11001; Thermo Fisher Scientific) and Hoechst 33342 (Thermo Fisher Scientific) for 1 h at 4°C. After incubation, the cells were washed with PBS and incubated with Alexa Fluor 555-conjugated anti-rabbit antibody (1:1,000 dilution, A21428; Thermo Fisher Scientific) for 1 h at room temperature. To simultaneously detect dsRNA, NP/NEP, and NS1, cells were incubated with Alexa Fluor 647-conjugated anti-NS1 mouse antibody (1:500 dilution, sc-130568 AF647; Santa Cruz Biotechnology, Dallas, TX, USA) for 1 h at room temperature subsequent to all incubation processes. Similarly, Alexa Fluor 647-conjugated anti-IRF3 mouse antibody (1:500 dilution, 566347; BD Pharmingen, San Diego, CA, USA) was used for quadruple staining. All the antibodies were diluted in PBS containing 10% Blocking One. Sectional images were captured and deconvoluted using a DeltaVision Elite system (GE Healthcare, Chicago, IL, USA) with a 60× oil-immersion objective on an Olympus IX71 microscope.

### Purification of vRNP

The influenza A virus A/Puerto Rico/8/34 (H1N1) (PR8) was prepared according to previously reported procedures (45). vRNPs were purified from PR8 virions using glycerol gradient ultracentrifugation as reported previously (29). The collected fractions were mixed with 2× Tris-glycine sodium dodecyl sulfate (SDS) sample buffer (Novex; Invitrogen) and subsequently subjected to SDS-PAGE on a 4%–15% Mini Protean TGX precast gel (Bio-Rad Laboratories, Hercules, CA, USA).

### Purification of dsRNA-vRNP complex from infected cells

A549 cells were infected with the FLAG–PB2 virus at an MOI of 0.1 and incubated in MEM/BSA at 37°C. At 24 hpi, cells were scraped from dishes in ice-cold PBS and subsequently pelleted through centrifugation at 780 × *g* for 10 min at 4°C. The resultant pellets were resuspended in lysis buffer (50 mM Tris-HCl [pH 8.0], 150 mM NaCl, 5 mM MgCl_2_, 10% glycerol, 0.05% NP-40, 2 mM dithiothreitol [DTT], 10 mM ribonucleoside-vanadyl complex [New England Biolabs, Beverley, MA, USA], and 1× complete EDTA-free protease inhibitor [Roche, Mannheim, Germany]). A rotation was implemented for 15 min at 4°C, followed by centrifugation at 20,000 × *g* for 15 min at 4°C. The resultant supernatant was incubated with anti-FLAG M2 affinity gel (Merck) on a rotator for 1 h at 4°C. The gels underwent a single wash with lysis buffer and three consecutive washes with wash buffer (50 mM Tris-HCl [pH 8.0], 200 mM NaCl, 50 mM Na_2_HPO_4_, and 2 mM DTT), subsequently eluting in wash buffer with 500 ng/µL FLAG peptide (Merck) through rotation on a rotator for 30 min at 4°C. The FLAG-tagged vRNP was further purified by glycerol gradient ultracentrifugation as reported previously (29). Subsequently, the cellular lysates, eluates, and purified fractions were subjected to electrophoresis on an SDS-polyacrylamide gel, followed by silver staining or immunoblotting using anti-NP mouse monoclonal antibody (1:10,000 dilution, ab20343; Abcam, Cambridge, UK) and anti-PB1 goat polyclonal antibody (1:10,000 dilution, sc-17601; Santa Cruz Biotechnology) as primary antibodies. The secondary antibodies used were horseradish peroxidase-conjugated anti-mouse (NA931; GE Healthcare) and anti-goat (ab6741; Abcam) antibodies.

### Expression and purification of recombinant NS1 protein

The NS1 protein fused with a His-tag was expressed in *E. coli* Rosseta (DE3) pLysS cells using the pET-14b plasmid. The *E. coli* transformants were cultured at 37°C in Luria-Bertani (LB) medium supplemented with 100 µg/mL of ampicillin. Upon reaching an optical density at 600 nm (OD_600_) of 0.8, 1 mM of isopropylthio-β-galactoside was introduced, and the cells were grown for an additional 4 h at 37°C. Following this, *E. coli* cells were harvested by centrifugation at 8,000 × *g* for 5 min and resuspended in binding buffer (20 mM Tris-HCl [pH 8.0], 500 mM NaCl, and 5 mM imidazole). The cells were lysed by sonication in an ice-cold water bath. Recombinant NS1 was purified using a Ni^2+^-immobilized column (His-Bind Purification Kit; Merck) according to the manufacturer’s instructions.

### *In vitro* RNA synthesis using virion-derived vRNPs

A concentration of 0.01 mg/mL of purified vRNP was subjected to incubation in 50 mM Tris-HCl buffer (pH 8.0) containing 5 mM MgCl_2_; 40 mM KCl; 1 mM DTT; 10 µg/mL actinomycin D; 1 mM each of ATP, CTP, GTP, and UTP; 1 U/µL RNasin Plus RNase inhibitor (Promega, Madison, WI, USA); and 1 mM ApG (IBA Lifesciences, Göttingen, Germany) at 30°C for 15 min. For the purpose of masking the dsRNA with NS1, 0.1 mg/mL of the purified recombinant NS1 was introduced into the *in vitro* transcribed vRNP mixture, followed by a 1 h incubation period on ice.

### HS-AFM

A 2 µL aliquot of the specimen was deposited onto freshly cleaved mica devoid of surface modification. Following incubation for 5 min at ambient temperature, the mica surface was thoroughly washed with imaging buffer (50 mM Tris-HCl [pH 8.0], 5 mM MgCl_2_, 40 mM KCl, and 1 mM DTT). The mica surface was submerged in a liquid chamber filled with imaging buffer for observation using an HS-AFM system (Nano Explorer; Research Institute of Biomolecule Metrology Co., Ltd., Ibaraki, Japan). HS-AFM was executed at room temperature in the tapping mode, wherein the cantilever oscillated vertically at its resonant frequency during lateral and vertical scanning of the cantilever chip, intermittently tapping the sample surface. Images were captured at a rate of two images per second using cantilevers featuring a 0.1 N/m spring constant and a resonance frequency in water of 0.6 MHz (BL-AC10DS; Olympus, Tokyo, Japan). To acquire high-resolution images, electron-beam-deposited tips (46) were used. The *in situ* observation of RNA digestion by RNase was executed through introducing a 0.2 U/µL of ShortCut RNase III (New England Biolabs) to the AFM liquid cell during imaging. To facilitate the detachment of the NS1 protein from the dsRNA, the specimen underwent prolonged observation, gradually diminishing the setpoint on the AFM system. A minimum of five independent experiments were conducted, and all HS-AFM images were viewed and analyzed using Kodec 4.4.7.39 (47). Individual images were subjected to a low-pass filter and flattening to eliminate spike noise and flatten the xy-plane, respectively.

### Statistics and reproducibility

All statistical analyses were conducted using the Prism 9.5.0 software (GraphPad, San Diego, CA, USA), with the specific methodology for each analysis delineated in the respective figure legends. A *P*-value of less than 0.05 was considered statistically significant. Each experiment documented in the manuscript was replicated at least three times, yielding consistent and reproducible results.

## Data Availability

All data substantiating the conclusions of this investigation are contained within the article and supplemental material or can be obtained from the corresponding authors upon judicious and reasonable request.

## SUPPLEMENTAL MATERIAL

Supplemental material jvi.00737-25-s0001.pdf Fig. S1 to S8, Tables S1 and S2, and legends for Movies S1 and S2.

Movie S1 jvi.00737-25-s0002.mp4 Digestion of the looped RNA by RNase III.

Movie S2 jvi.00737-25-s0003.mp4 Detachment of NS1 from the looped RNA.

ASM does not own the copyrights to Supplemental Material that may be linked to, or accessed through, an article. The authors have granted ASM a non-exclusive, world-wide license to publish the Supplemental Material files. Please contact the corresponding author directly for reuse.
