# Supplementary material for: Influenza A virus circumvents the innate immune response through the sequestration of double-stranded RNA
Source: J Virol. 2025 Sep 8;99(10):e00737-25. doi: 10.1128/jvi.00737-25 (PMC12548412; doi:10.1128/jvi.00737-25)
Supplement: Supplemental material — Fig. S1 to S8, Tables S1 and S2, and legends for Movies S1 and S2. [file jvi.00737-25-s0001.pdf]

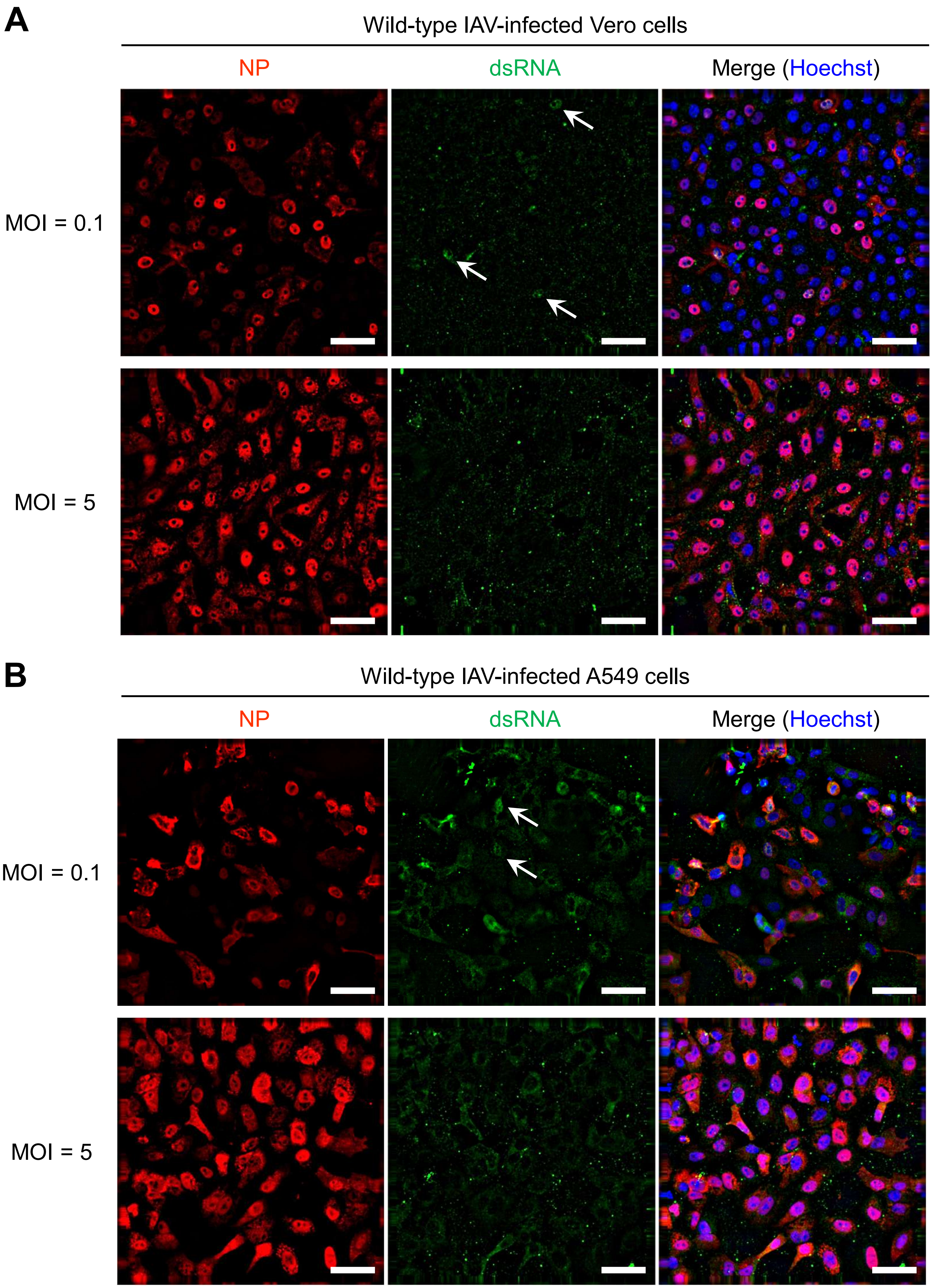

Fig. S1. Effect of MOI on IAV dsRNA detection.

Vero (A) and A549 (B) cells were infected with the IAV WSN strain at MOIs of 0.1 or 5 and subsequently fixed at 10 and 14 hpi, respectively. The presence of NP and dsRNA in infected cells was detected by IFA using anti-NP and anti-dsRNA antibodies, respectively. All dsRNA-positive cells within a given viewpoint are indicated by arrows. Hoechst staining was used to stain cellular nuclei. The scale bars represent 50 μm.

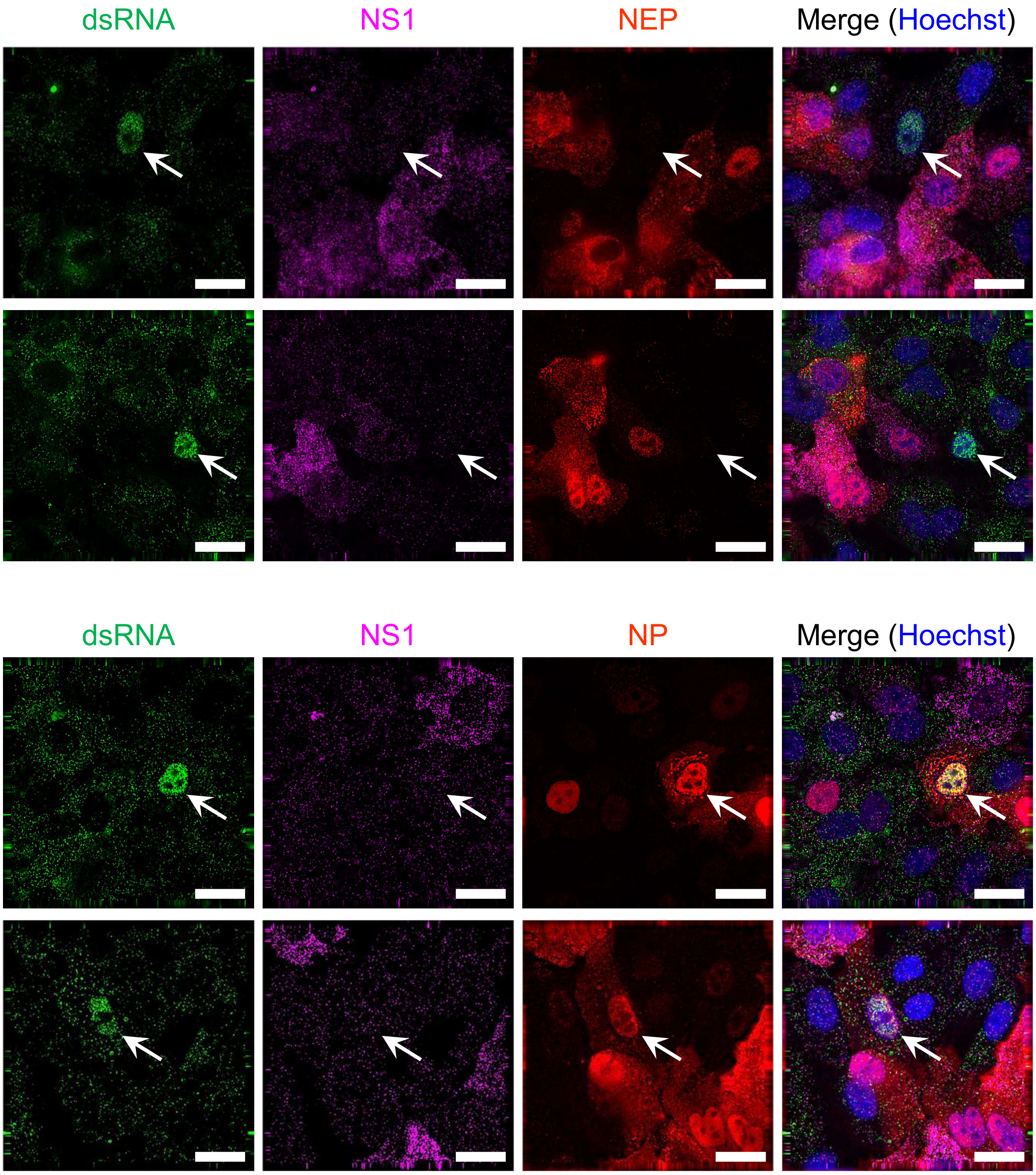

Fig. S2. Simultaneous detection of NS1 and NEP in dsRNA-positive cells.
A549 cells were infected with the IAV WSN strain at an MOI of 0.1 and subsequently fixed at 14 hpi. NS1 and NEP expression in dsRNA-positive cells were concomitantly detected by IFA using anti-NS1, anti-NEP, and anti-dsRNA antibodies (upper panels). To confirm NP expression in dsRNA-positive cells, NS1 and NP were simultaneously detected using anti-NP antibodies (lower panels). Cell nuclei were stained with Hoechst. Arrows indicate infected cells producing dsRNAs. For each panel, two representative image sets (top and bottom) from a single experiment are shown. The experiment was independently performed at least three times with similar results. The scale bars denote 20 μm.

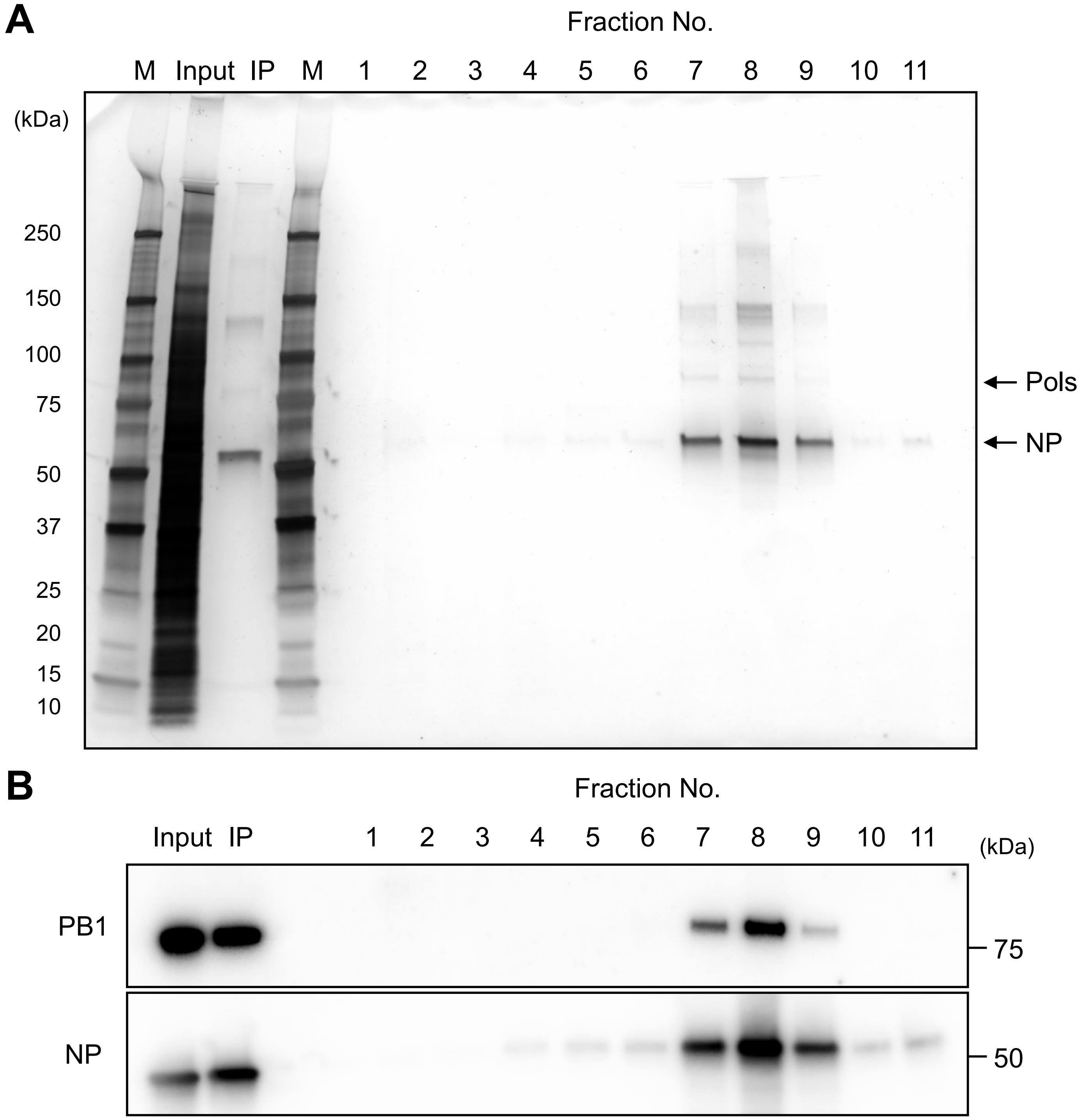

Fig. S3. Isolation of the vRNP–looped RNA complex from cells infected with FLAG–PB2 virus. vRNPs purified from FLAG–PB2 virus-infected A549 cells were subjected to SDS-PAGE followed by silver staining (A) and western blotting (B). The supernatant from cell lysis (Input), immunoprecipitate eluted with the FLAG peptide (IP), and purified fractions following ultracentrifugation (Nos.1–11) were examined. In panel A, bands corresponding to RNA polymerases (Pols) and NP are indicated by arrows. The verification of vRNP purification was conducted in panel B using anti-PB1 and anti-NP antibodies. Molecular weight marker proteins (M) were used as reference proteins. Uncropped gel and blot images are shown in Fig. S8.

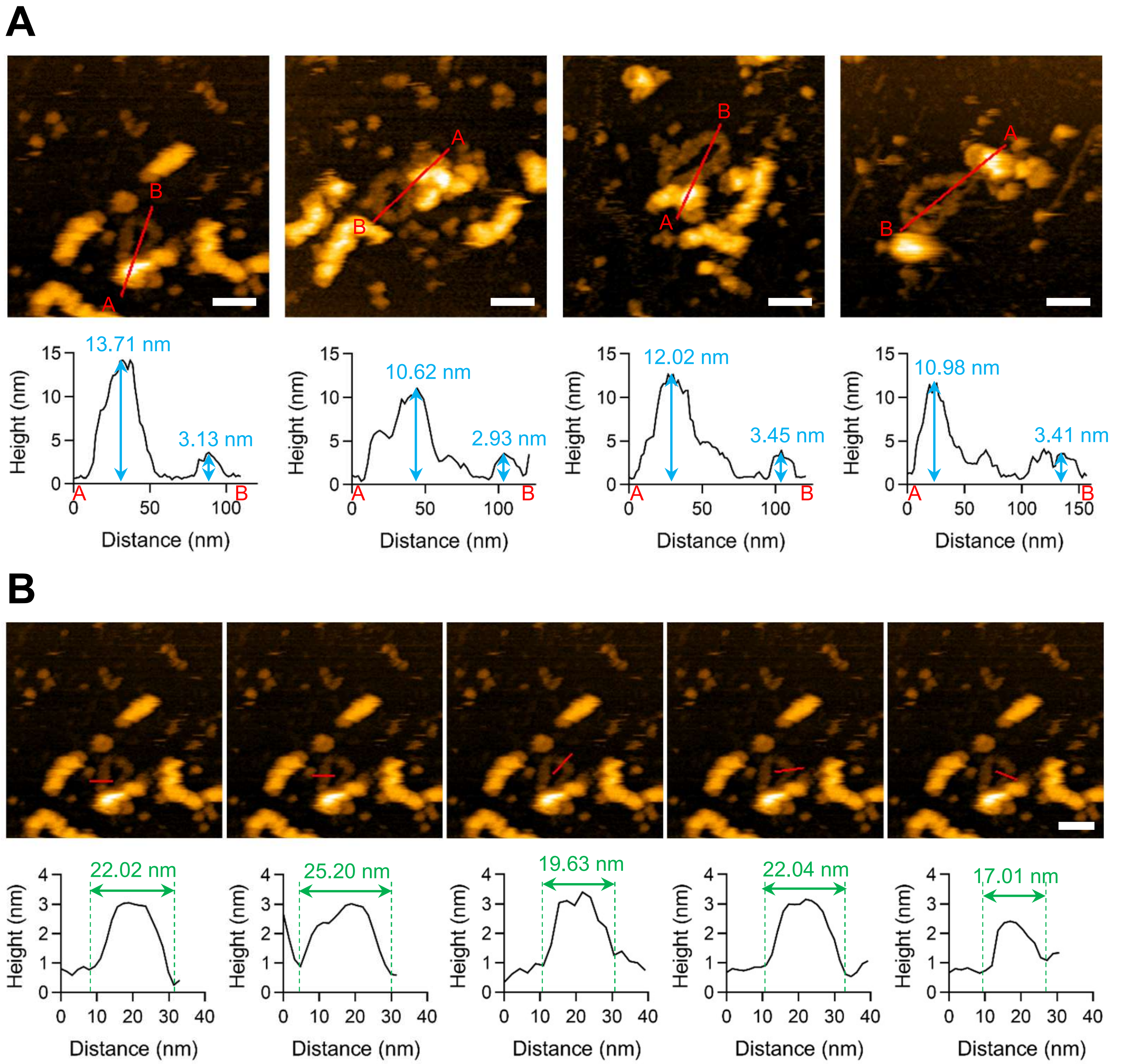

Fig. S4. Cross-sectional analysis of the looped structure associated with vRNP.
(A) Determination of the height of the vRNP and looped structures. A cross-sectional analysis was performed along the delineated red lines in the HS-AFM images (upper panels). The quantified heights of both vRNPs and looped structures are shown in the lower panels. (B) Quantification of the width of the looped structure. HS-AFM images analogous to that in Fig 3G (+NS1) are presented, and a cross-sectional analysis was conducted at distinct positions along the looped structure, as denoted by red lines. The measured widths are presented in the lower panels. All scale bars denote 50 nm.

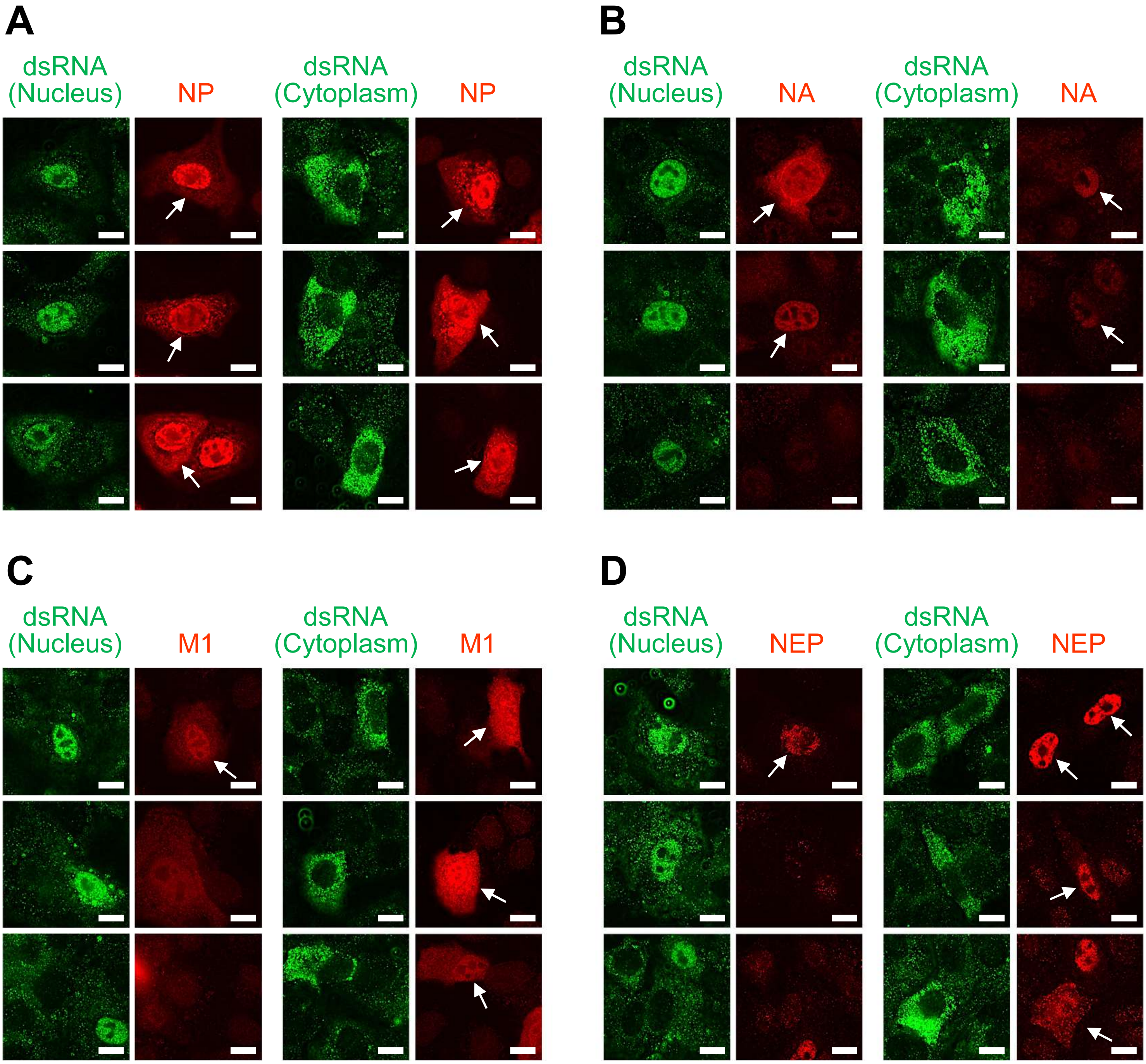

Fig. S5. Association between the localization of dsRNA and viral protein expression in ΔNS1 virus-infected cells.

A549 cells were infected with ΔNS1 virus at an MOI of 0.1 and subsequently fixed at 24 hpi. dsRNA was detected by IFA using anti-dsRNA antibodies. Viral protein expression was examined using anti-NP (A), anti-NA (B), anti-M1 (C), and anti-NEP (D) antibodies. Cells exhibiting dsRNA production in the nucleus and cytoplasm, from different fields of view in a single experiment, are shown in the left and right panels, respectively. Arrows indicate cells producing dsRNA and concurrently expressing the designated viral proteins. For each panel, three representative image sets (top, middle, and bottom) from a single experiment are shown. The experiment was independently repeated at least three times with comparable results. Scale bars represent 10 μm.

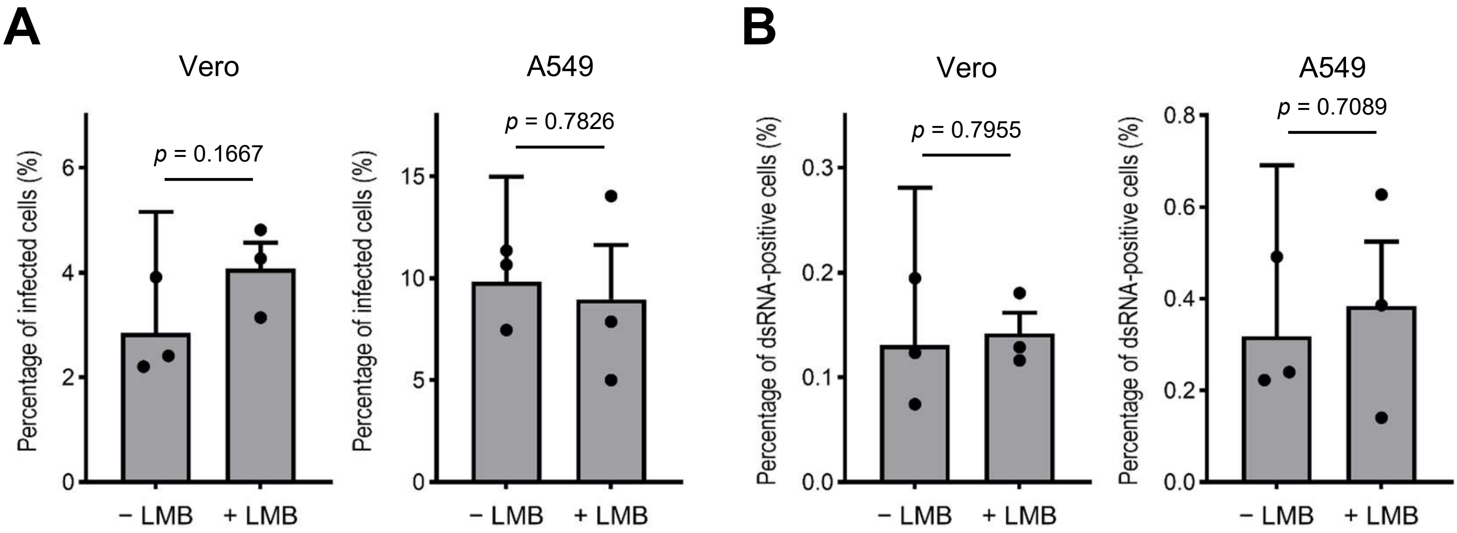

Fig. S6. Effect of LMB on virus infection and dsRNA detection.
Vero (left panel) or A549 cells (right panel) were infected with ΔNS1 virus at an MOI of 0.1, followed by incubation with or without LMB, and subsequently fixed at 24 hpi. NP-positive and dsRNA positive cells were detected and quantified by IFA. The proportions of NP-positive cells and dsRNA-positive cells relative to the total observed cells are depicted in panels A and B, respectively. Data represent the mean ± 95% confidence intervals derived from three biologically independent experiments. Statistical significance was assessed using an unpaired *t*-test.

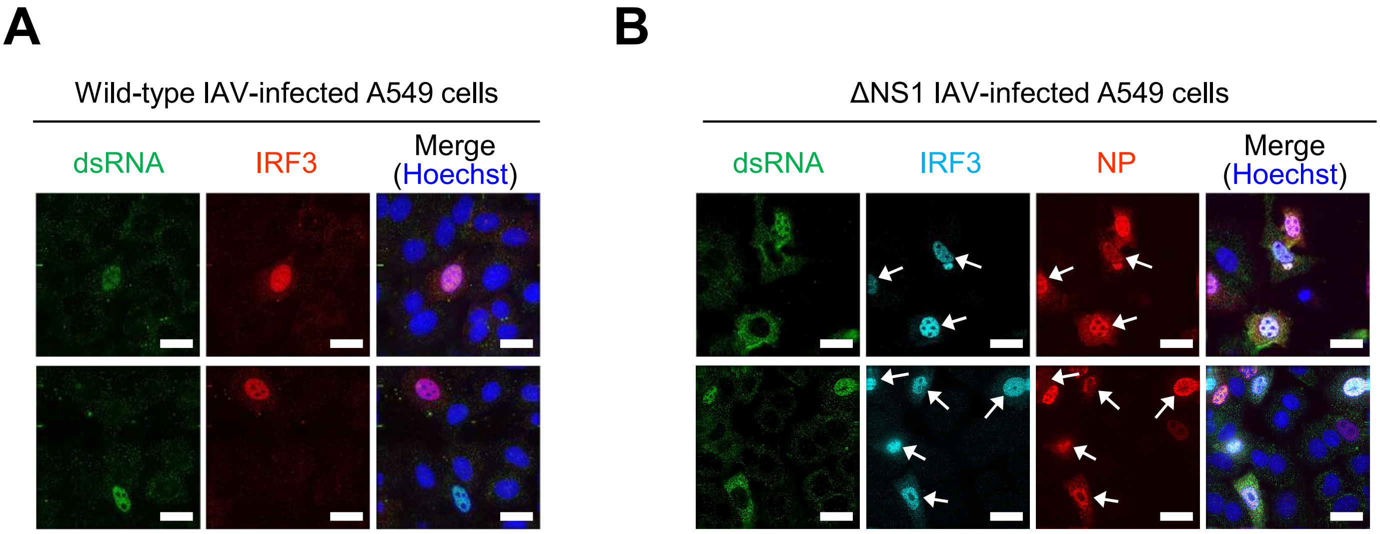

Fig. S7. dsRNA production and IRF3 nuclear translocation in infected cells.
(A) A549 cells were infected with the wild-type IAV WSN strain at an MOI of 0.2 and were subsequently fixed at 24 hpi. IFA was conducted to detect dsRNAs using anti-dsRNA antibodies. To examine the nuclear translocation of IRF3, anti-IRF3 antibodies were applied. Nuclear translocation of IRF3 was observed in certain dsRNA-positive cells (upper panels), whereas the other cells did not exhibit nuclear translocation of IRF3 (lower panels). Cell nuclei were stained with Hoechst. (B) A549 cells were infected with ΔNS1 virus at an MOI of 0.1 and were subsequently fixed at 24 hpi. NP expression and IRF3 nuclear translocation in dsRNA-positive cells were simultaneously detected by IFA using anti-NP, anti-IRF3, and anti-dsRNA antibodies. Arrows indicate nuclei exhibiting IRF3 nuclear translocation. Two representative image sets (top and bottom) from a single experiment are shown. The experiment was independently repeated at least three times with similar results. All scale bars represent 20 μm.

A

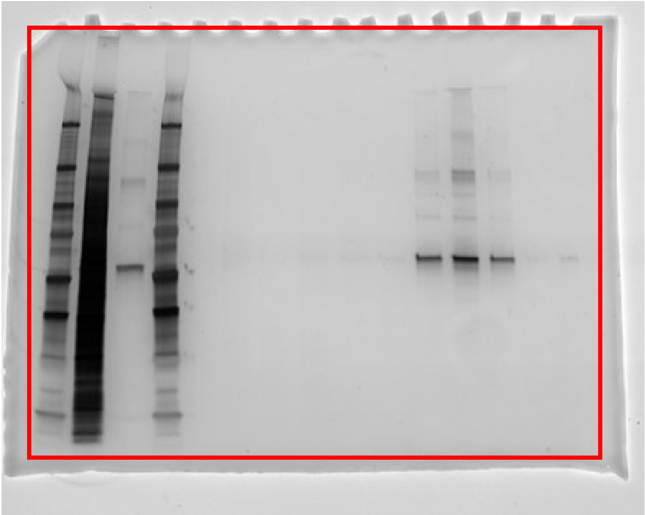

B

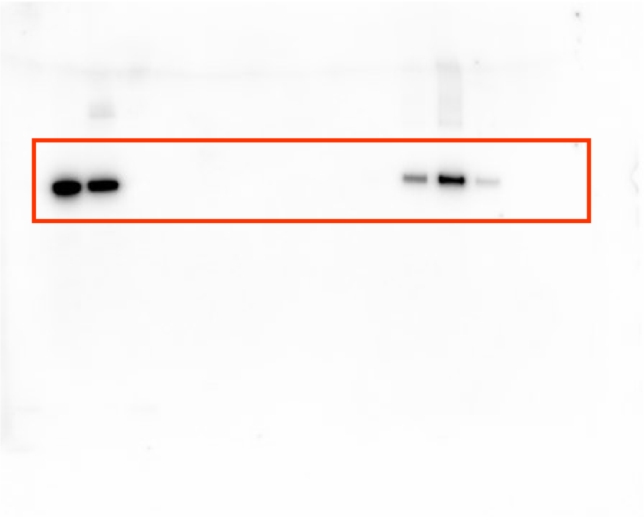

C

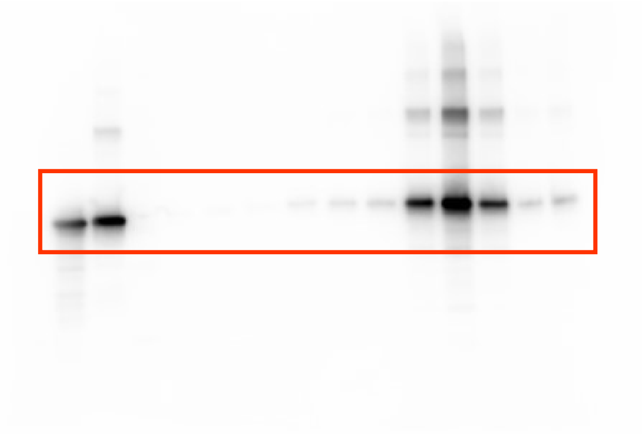

D

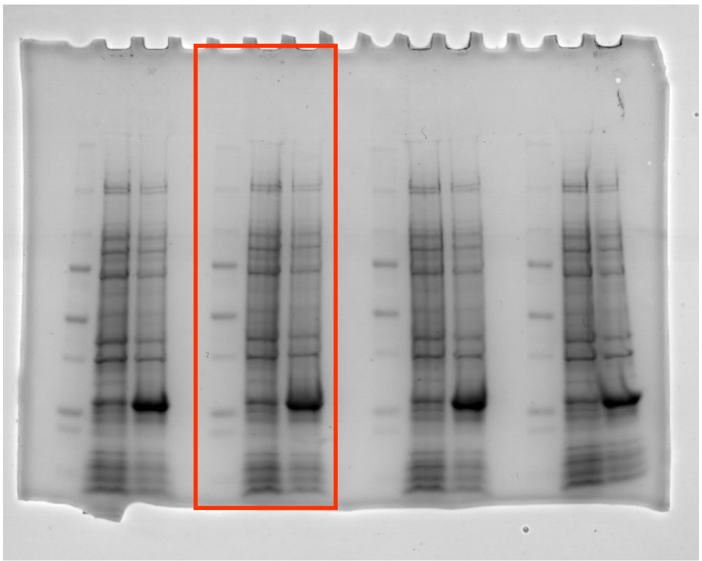

E

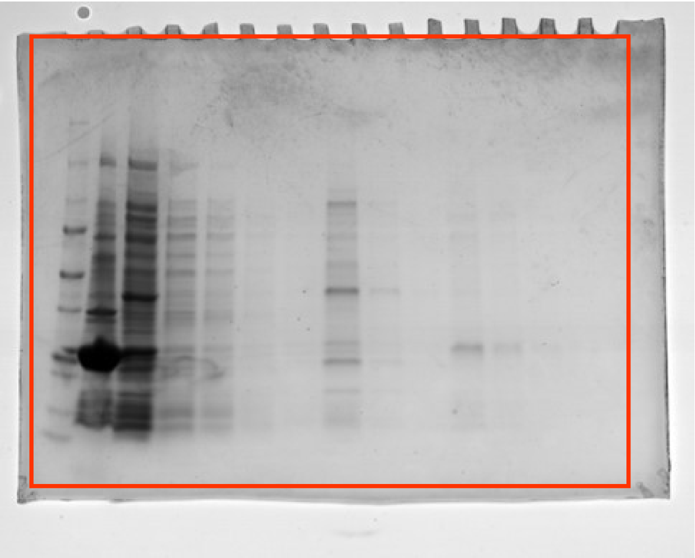

Fig. S8. Uncropped gel and blot images.
(A) Uncropped gel image for Fig. S3A. (B) Uncropped blot image for Fig. S3B, upper panel. (C) Uncropped blot image for Fig. S3B, lower panel. (D) Uncropped gel image for Fig. 3F, left panel. (E) Uncropped gel image for Fig. 3F, right panel.

Table S1. Number of dsRNA-positive cells infected with wild-type IAV WSN strain at different MOI.

Cell	Number of dsRNA-positive cells*		
	Mock	MOI = 0.1	MOI = 5
Vero (10 hpi)	7<br>(2/3/2)	92<br>(16/44/32)	5<br>(0/3/2)
A549 (14 hpi)	1<br>(1/0/0)	87<br>(36/22/29)	0<br>(0/0/0)

*dsRNA-positive cells per culture dish were counted for three distinct samples (each number is shown in parentheses) and total numbers are presented in this table.

Table S2. Number of viral protein-expressing and dsRNA-positive A549 cells infected with wild-type IAV WSN strain at MOI of 0.1.

Antibody	Viral protein-expressing A549 cells / dsRNA-positive A549 cells*			
	Expt. #1	Expt. #2	Expt. #3	Total
α-NP	35/36	21/22	29/29	85/87 (97.7%)
α-PA	19/22	26/29	21/27	66/78 (84.6%)
α-NA	8/18	13/23	15/31	36/72 (50.0%)
α-NS1	4/31	4/43	4/34	12/108 (11.1%)
α-NEP	1/33	3/30	1/27	5/90 (5.6%)

*The number of dsRNA-positive cells per culture dish was counted, and the expression of viral protein in each cell was examined. The percentage of dsRNA-positive cells expressing each viral protein was calculated from three independent experiments (Expt. #1–3) and shown in parentheses.

Movie S1. Digestion of the looped RNA by RNase III.
RNase III was added during HS-AFM observation of looped RNA–vRNP complex. Scan area: 300×300 nm^2. Observation period: 90 sec.

Movie S2. Detachment of NS1 from the looped RNA.
The looped structure observed after NS1 addition was observed over an extended duration using HS-AFM with applying an augmented force. Scan area: 300×300 nm^2. Observation period: 450 sec.
